# Melanocortin System in Kidney Homeostasis and Disease: Novel Therapeutic Opportunities

**DOI:** 10.3389/fphys.2021.651236

**Published:** 2021-02-24

**Authors:** Mingyang Chang, Bohan Chen, James Shaffner, Lance D. Dworkin, Rujun Gong

**Affiliations:** Division of Nephrology, Department of Medicine, University of Toledo College of Medicine, Toledo, OH, United States

**Keywords:** melanocyte-stimulating hormone, adrenocorticotropic hormone, pro-opiomelanocortin, glomerulus, kidney disease, melanocortin receptors

## Abstract

Melanocortin peptides, melanocortin receptors, melanocortin receptor accessory proteins, and endogenous antagonists of melanocortin receptors are the key components constituting the melanocortin hormone system, one of the most complex and important hormonal systems in our body. A plethora of evidence suggests that melanocortins possess a protective activity in a variety of kidney diseases in both rodent models and human patients. In particular, the steroidogenic melanocortin peptide adrenocorticotropic hormone (ACTH), has been shown to exert a beneficial effect in a number of kidney diseases, possibly via a mechanism independent of its steroidogenic activity. In patients with steroid-resistant nephrotic glomerulopathy, ACTH monotherapy is still effective in inducing proteinuria remission. This has inspired research on potential implications of the melanocortin system in glomerular diseases. However, our understanding of the role of the melanocortinergic pathway in kidney disease is very limited, and there are still huge unknowns to be explored. The most controversial among these is the identification of effector cells in the kidney as well as the melanocortin receptors responsible for conveying the renoprotective action. This review article introduces the melanocortin hormone system, summarizes the existing evidence for the expression of melanocortin receptors in the kidney, and evaluates the potential strategy of melanocortin therapy for kidney disease.

## Introduction

**FIGURE 1:**
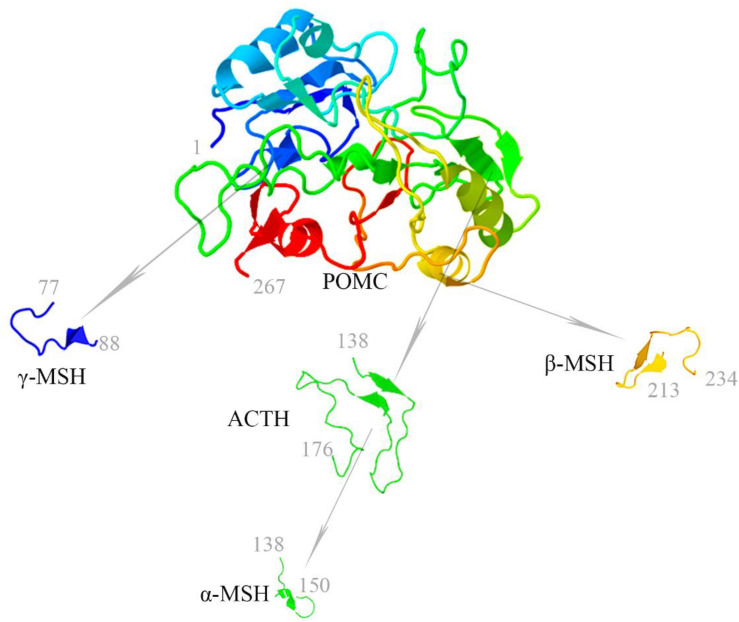
Melanocortin peptides (ACTH, α-MSH, β-MSH, and γ-MSH) are all derived from the common precursor protein pro-opiomelanocortin (POMC). The 3-dimensional structure modeling of human melanocortin peptides was generated based on amino acid sequences by using the following two servers for automated protein structure modeling (http://protein.ict.ac.cn/FALCON/#focus; http://bioserv.rpbs.univ-paris-diderot.fr/services/PEP-FOLD/).

The melanocortin system is a neuroimmune endocrine hormone system that encompasses five melanocortin receptors (MC1R∼MC5R), four pro-opiomelanocortin (POMC)-derived melanocortin peptides (ACTH, α-MSH, β-MSH, and γ-MSH) (Figure 1), endogenous antagonists, i.e., agouti-signaling protein (ASP or ASIP) and agouti-related protein (AGRP), and melanocortin receptor accessory proteins (MRAP) (Table 1) (Gantz and Fong, 2003). In the early 20th century, Atwell (1919) found that there might be some substances in the pituitary gland that darkens skin color. They implanted pituitary extracts into frogs that became albino after pituitary removal, and found that the frogs regained pigmentary responses (Atwell, 1919). The underlying mechanism has been elusive until about 60 years later after the development of the amino acid sequences and solid-phase synthesis of synthetic polypeptides. It was finally confirmed that melanocortin peptides were derived from a common precursor molecule, which earned its name POMC due to its opiate derivative β-endorphin (Eipper and Mains, 1980).

**TABLE 1:** Constituent components of the melanocortin hormone system.

The melanocortin hormone system			
Ligands	Melanocortin receptors	Melanocortin antagonists	Melanocortin receptor accessory proteins
ACTH, α-MSH, β-MSH, and γ-MSH	MC1R, MC2R, MC3R, MC4R, and MC5R	Agouti, ASIP, and AGRP,	MRAP1, MRAP2, mahogany protein, and syndecan-3

*ASIP, agouti-signaling protein; AGRP, agouti-related protein; MRAP, melanocortin receptor accessory protein.*

The melanocortin hormone system exerts a diverse array of physiological functions, including pigmentation, adrenocortical steroidogenesis, energy homeostasis, natriuresis, erectile response, exocrine gland secretion, analgesia, inflammation, immunomodulation, and temperature control (Cone, 2006). A number of melanocortin peptides and small molecule melanocortin mimetics that are able to potentiate or mitigate these functions are undergoing preclinical testing or clinical trial. Among these, ACTH has been used since 1952 as Food and Drug Administration (FDA)-approved first line therapy for a myriad of diseases, including nephrotic syndrome. Mechanistically, the use of ACTH was primarily based on its ability to increase the adrenal production of glucocorticoids. As such, its use was later substituted by synthetic corticosteroids. However, a growing body of evidence recently suggests that ACTH monotherapy is able to effectively alleviate steroid-resistant nephrotic syndrome, denoting that ACTH achieves its renal protection via mechanisms beyond adrenocortical steroidogenesis (Gong, 2011). Given that multiple kidney cells express MCRs, it is posited that the kidney may be a quintessential effector organ of the melanocortin hormone system and that melanocortin peptides may directly target diverse types of kidney cells to convey a renoprotective activity.

## The Melanocortin Hormone System

### Melanocortin Receptors

The five melanocortin receptors (MCRs, MC1R∼MC5R) are expressed in a diverse array of tissues and belong to the guanine nucleotide-binding protein-coupled receptor (GPCR) family. MCRs have different affinities to agonists and antagonists causing a vast array of physiological effects (Gong, 2014). As opposed to their 7-transmembrane GPCR counterparts whose primary function is the intracellular induction of cyclic adenosine monophosphate (cAMP), MCRs can also activate the inositol triphosphate pathway (Konda et al., 1994) and the protein kinase C (PKC) pathway (Kapas et al., 1995), of which the downstream functions have not yet been fully elucidated. In addition, some MCRs signal through guanine nucleotide-dependent and independent mechanisms and their functional coupling to agonists at the cell surface is regulated by interacting accessory proteins, like MRAPs and β-arrestins (Rodrigues et al., 2015). It is worth noting that MCRs have phylogenetic differences, as reported by Logan et al. (2003) that there are six types of MCRs (including two MC5R orthologs) in Zebrafish whereas only four types of MCRs (lacking MC3R) in Fugu.

**FIGURE 2:**
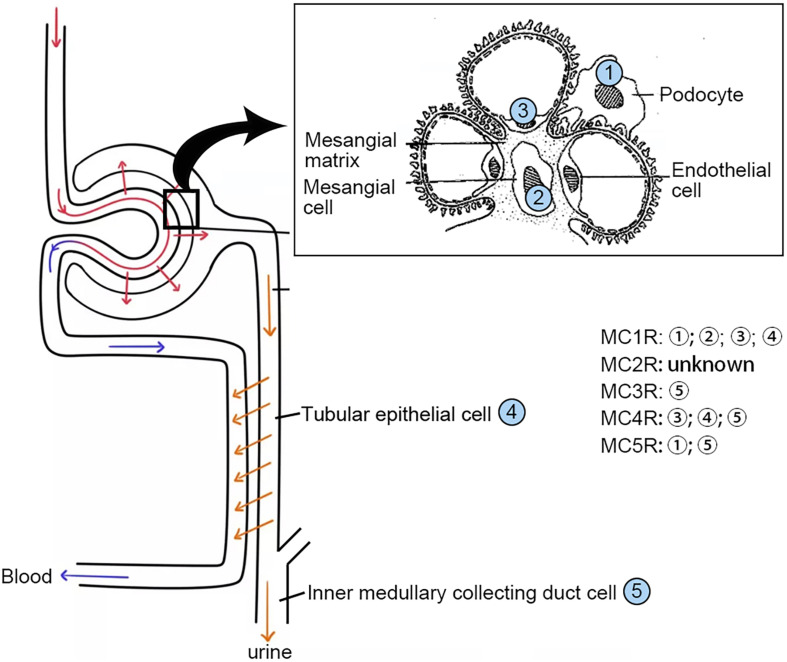
Distribution of the MCRs in kidney cells. MCRs are widely expressed in renal parenchymal cells. MCR, melanocortin receptor.

MC1R is expressed abundantly in skin cells where it is a key control point in determining skin and hair pigmentation (Jackson et al., 2007). In support of this, loss-of-function or null mutations in MC1R are associated with a switch from eumelanin to phaeomelanin production, resulting in red hair color, freckles, and fair skin (Fitzpatrick skin type 1) in humans and yellow coat color in mice. MC1R was originally named MSH-R before Mountjoy et al. (1992) completed the cloning of the human MSH receptor, and later the other four distinct MSH receptors, using the cDNA library prepared from melanoma. MC1R is also widely expressed in other tissues including adrenal gland, kidney (Figure 2), lung, brain, lymph nodes, spleen, and leukocytes where it plays a key role in regulating inflammatory reaction and immune response (Cone et al., 1996; Lindskog et al., 2010). Indeed, as compared with wild-type mice, dextran sodium sulfate or Citrobacter rodentium-induced colitis significantly was aggravated in MC1R null (MC1R^*e/e*^) mice that have a frameshift mutation between exon 4 and 5 and are lacking a functional MC1R (Maaser et al., 2006). Moreover, atherosclerosis in the aortic sinus and in the whole aorta caused by apolipoprotein E deficiency plus high-fat diet was exacerbated in recessive yellow (Mc1re/e) mice, associated with an enhanced arterial recruitment of Ly6Chigh monocytes (Rinne et al., 2018).

MC2R is predominantly expressed in adrenal cortices and adipose tissues. MC2R is unique that it only binds to ACTH, which is produced by the pituitary gland and circulated to the adrenal cortex where MC2R mediates steroidogenesis. In adipose tissues, the MC2R is known to mediate lipolysis (Cone et al., 1996). Mutations in human MC2R are associated with familial glucocorticoid deficiency (FGD), which manifests with low levels of cortisol and high levels of ACTH due to the impairment in cortisol-mediated negative feedback to the hypothalamic release of corticotropin-releasing factor and to the pituitary release of ACTH (Ramachandrappa et al., 2013).

MC3R is expressed mainly in the brain, kidney, and other periphery tissues. It has the same affinity to ACTH as other types of MCRs. While the exact pathobiological functions of MC3R still remain enigmatic, more and more evidence suggests that MC3R may be involved in energy homeostasis (Ghamari-Langroudi et al., 2018). In support of this, MC3R knockout mice have increased fat mass, decreased lean mass, hyperphagia, less activity, and mild late-onset obesity. Moreover, experimental models of cachexia in the MC3R knockout mouse are more susceptible to body weight loss. However, MC3R gene polymorphism in humans has not yet been definitively associated with obesity (Mencarelli et al., 2011).

MC4R is largely expressed in the central nervous system, where some MC4R expression overlaps with the localization of MC3R (Ghamari-Langroudi et al., 2018). Its function is believed to be regulation of energy homeostasis and food intake (Huszar et al., 1997). Targeted deletion of MC4R gene is associated with early-onset severe obesity, hyperphagia, and hyperinsulinemia. Even in cachexia models, MC4R knockout mice still have normal food intake, growth, and activity as compared with wild-type mice. Furthermore, the study of MC4R knockout heterozygotes and homozygotes demonstrated that body weight homeostasis is more severely affected in homozygotes than in heterozygotes, consistent with a gene dosage effect (Cone, 2006).

MC5R is highly expressed in exocrine glands and peripheral tissues including, but not limited to, the adrenal gland, adipose tissue, kidney, and leukocytes. Mice lacking MC5R were found to have a severe defect in water repulsion and thermoregulation due to decreased production of sebaceous lipids. This finding may have implications for future research and treatment of skin disorders, such as acne and dermatitis (Chen et al., 1997). Other than the regulation of exocrine glands, MC5R may also play an important role in farnesene-stimulated aggression in rodents, as well as in anti-inflammation and immunomodulation (Gong, 2014). Nevertheless, the exact function of MC5R in many tissues remains unclear.

### Agonists and Antagonists

The melanocortins include ACTH and the three melanocyte-stimulating hormones, namely α-MSH, β-MSH, and γ-MSH. Among these hormones, α-MSH is the most potent agonist for all MCRs except for MC2R, which is activated only by ACTH. Furthermore, the β- and γ-MSH bind to MC3R with the same affinity as that of α-MSH (Kirwan et al., 2018). All melanocortin peptides, including MSH and ACTH, share a conserved core tetrapeptide sequence His-Phe-Arg-Trp, which is essential to recognize and bind to MCRs. However, this sequence is not sufficient for binding to MC2R. Another tetrapeptide motif Lys-Lys-Arg-Arg is additionally required as the address sequence that permits MC2R recognition and is unique to ACTH.

ASIP and AGRP are endogenous antagonists of MCRs, and play crucial roles, respectively, in the regulation of pigmentation and energy balance. ASIP is the human homolog of the mouse agouti gene and encodes a 132 amino acid protein. ASIP is widely expressed in diverse human tissues, including the adipose tissue, testis, ovary, heart, kidney, and liver, denoting a diverse range of functions (Voisey et al., 2003). In the rodents, ASIP, encoded by the *agouti* gene, is primarily expressed in the skin, where it competes with MSH to bind with MC1R, resulting in a decreased intracellular cAMP induction and thus affecting coat color. ASIP is able to bind to all five MCRs with the highest affinity for MC1R and MC4R (Cortes et al., 2014). In the hypothalamus, ectopic expression of *agouti* causes obesity due to its antagonism of MC4R. AGRP is found in the adrenal gland, kidney, lung, and plasma (Shen et al., 2002), but is mainly expressed in the hypothalamus, where it acts as an antagonist to MC3R and MC4R (Voisey et al., 2003). Interestingly, over-expression of AGRP or administration of exogenous AGRP stimulates feeding, leading to obesity. However, unlike ASIP, there are no changes in pigmentation because AGRP doesn’t bind to MC1R (Shen et al., 2002).

### Melanocortin Receptor Accessory Proteins (MRAPs)

The activity of MCRs is precisely regulated, not only by their ligands (melanocortins or antagonists), but also by other accessory proteins, named MRAP. MRAP is a single-pass transmembrane protein consisting of anti-parallel homodimers and plays an important role in the regulation of trafficking or signaling of the 5 MCRs (Kim et al., 2014). MRAP exists in 2 isoforms, i.e., MRAP1 and MRAP2. MRAP1 is expressed in very few tissues like adipocytes and the adrenal glands, where it is essential for proper trafficking and signaling of the MC2R. In contrast, MRAP2 is widely expressed in a myriad of tissues in addition to the adrenal glands. This distinct pattern of expression may explain why mutations of MRAP1 account for 15–20% FGD (Rodrigues et al., 2015). MRAPs have no effect on the trafficking of MC1R and MC3R but reduce surface expression of MC4R and MC5R (Sebag and Hinkle, 2007, 2010; Chan et al., 2009). Indeed, it appears that MRAP2 may act as a competitive inhibitor to MRAP1, but its exact role is still unclear. Mutations of MRAP2 are associated with obesity but it has not been found to be linked to FGD as MRAP1, suggesting that MRAP1 and MRAP2 are not functionally interchangeable (Kim et al., 2014). Besides MRAPs, a number of additional proteins also serve as potential accessory proteins for MCRs. For instance, the mahogany protein and syndecan-3 can, respectively, modulate the function of ASIP and AGRP via promoting their competition with MSH for MCRs. In addition, some endoplasmic reticulum-resident chaperones, like glucose-regulated protein (Yoon et al., 2018), have been shown to promote trafficking and the intracellular signal of MCRs (Rodrigues et al., 2015). The molecular mechanism underlying the interaction between MCRs and the specific accessory proteins warrants further research (Gantz and Fong, 2003).

**FIGURE 3:**
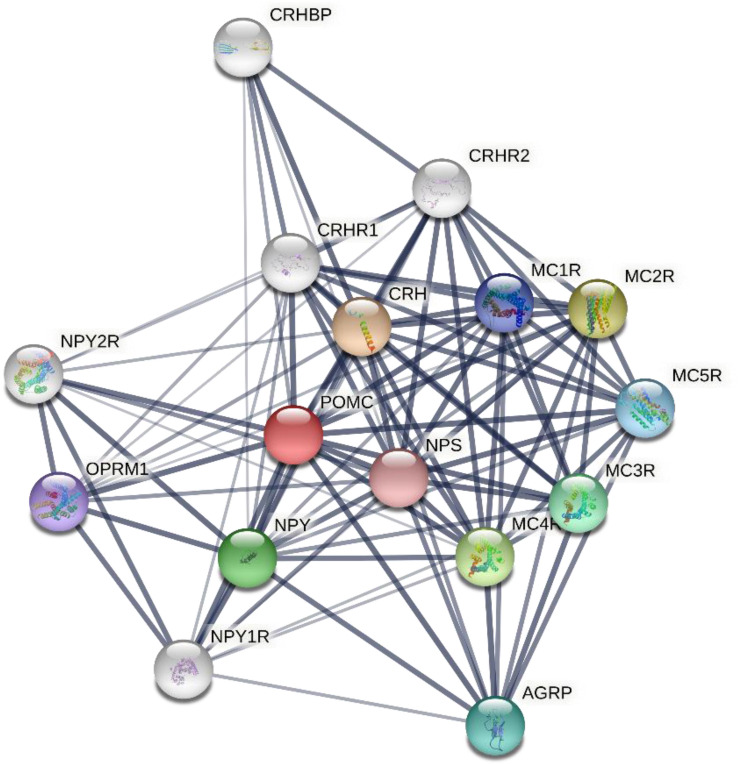
Protein-protein interactions network involving human melanocortin system and other key proteins as revealed by the STRING (Search Tool for the Retrieval of Interacting Genes/Proteins), a biological database and web resource of known and predicted protein–protein interactions. The associations between key proteins are indicated by the connecting lines. The thickness of the connecting lines represents the strength of the associations. The associations do not necessarily suggest that the proteins are physically binding each other, but may jointly contribute to a shared function. The analysis was performed using STRING V11.0 (STRING https://string-db.org/). CRH, corticotropin releasing hormone; NPY, neuropeptide Y; NPS, neuropeptide S; AGRP, agouti related protein homolog; OPRM1, mu-type opioid receptor; CRHBP, corticotropin-releasing factor-binding protein; MCR, melanocortin receptor.

In an effort to reveal the functional association between the melanocortin system and other key pathways, a protein interaction network diagram was constructed using Search Tool for the Retrieval of Interacting Genes/Proteins (STRING), a biological database and web resource of known and predicted protein-protein interactions, based on the search of the protein-protein interaction network database and functional enrichment analyses. These analyses are capable of determining the relationship of the melanocortin system with other proteins located in a network hub. The protein POMC was located in the most central area of the network, followed by corticotropin releasing hormone (CRH), corticotropin releasing hormone receptor (CRHR), neuropeptide Y (NPY), Neuropeptide Y receptor (NPYR), neuropeptide S (NPS), agouti related protein homolog (AGRP), mu-type opioid receptor (OPRM1), corticotropin-releasing factor-binding protein (CRHBP), and MCRs (Figure 3).

## The Role of Melanocortin System in Kidney Pathophysiology

The key physiologic function of the kidney is to filtrate and excrete metabolic waste/toxins and to regulate fluid and electrolyte homeostasis and acid-base balance. The glomerular filtration barrier (GFB) is instrumental for plasma filtration and consists of a highly specialized blood filtration interface that exhibits a high permeability to small and midsized solutes in plasma but retains relative impermeability to macromolecules. The GFB is made up of 3 anatomical layers known as the fenestrated capillary endothelium, glomerular basement membrane, and podocytes, respectively. The podocyte, a terminally differentiated epithelial cell, plays a vital role in controlling the permselectivity of the GFB, and its dysfunction is centrally implicated in various glomerular diseases (Haraldsson and Jeansson, 2009).

**FIGURE 4:**
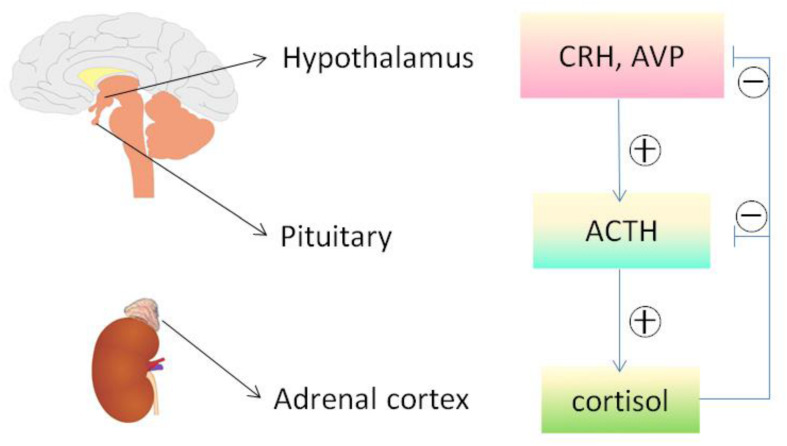
The hypothalamic-pituitary-adrenal axis. Cortisol released by the adrenal cortex in response to ACTH stimulation can negatively regulate the hypothalamus and pituitary, reduce the secretion of both corticotropin releasing hormone (CRH) and arginine vasopressin (AVP), and directly inhibit the pituitary production of ACTH and other melanocortins. The release of ACTH and other melanocortins is also affected by other factors via HPA axis, including stress and circadian rhythms. ACTH, adrenocorticotropic hormone; HPA, hypothalamic– pituitary–adrenal.

Adrenocorticotropic Hormone was approved in 1952 by the U.S. FDA for the treatment of nephrotic syndrome. At that time, ACTH was believed to act mainly via adrenocortical steroidogenesis, and thus had been widely used in the treatment of a number of inflammatory or autoimmune disorders, including rheumatoid arthritis (RA), gout, lupus, rheumatic fever, psoriasis, and ulcerative colitis (Gallo-Payet, 2016). However, due to its injectable route of administration and high cost, ACTH was later replaced by synthetic glucocorticoids, which are much more affordable and provide the convenient oral route of administration. Recently, a growing body of evidence indicates that ACTH is likely distinct from glucocorticoids in terms of clinical effectiveness as well as adverse effect profiles (Getting et al., 2002; Ko et al., 2004; Zaidi et al., 2010; Montero-Melendez, 2015). For instance, one of the serious complications of glucocorticoid treatment is the risk of osteoporosis and osteonecrosis. In stark contrast, patients with ACTH-producing adenomas typically do not develop osteonecrosis (Ko et al., 2004) despite having very high levels of circulating glucocorticoid secondary to over-activation of the Pituitary-Adrenal hormone axis (Figure 4). By using a rabbit model of steroid-induced avascular necrosis of the femoral head, Zaidi et al. (2010) demonstrated that ACTH therapy actually protects against osteoporosis and osteonecrosis. In addition, Getting et al. (2002) found in experimental models of gouty arthritis that ACTH treatment has a potent anti-inflammatory effect without altering circulating corticosterone levels and even in adrenalectomized rats. When examining all of the data, it appears that ACTH confers a better therapeutic efficacy but fewer side effects than glucocorticoids (Montero-Melendez, 2015), entailing that some steroidogenic-independent mechanisms may contribute to the unique beneficial effect of ACTH.

**FIGURE 5:**
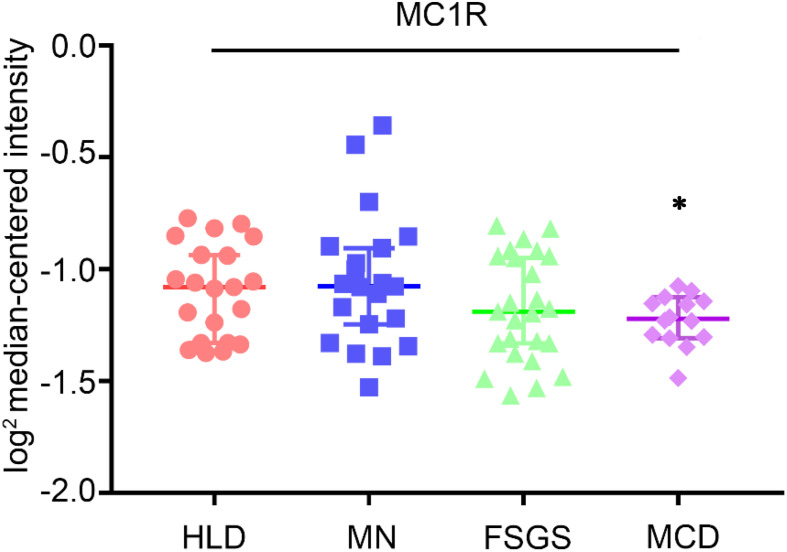
Expression levels of MC1R mRNA in glomeruli procured from healthy living donors and patients with diverse glomerular disease. Data were derived from www.Nephroseq.org on the basis of the Ju CKD Glom dataset. Glomerular expression of MC1R mRNA is not altered in glomerular diseases. Minimal Change Disease vs. Healthy Living Donor, ^∗^*P*-value: 0.011. Focal Segmental Glomerulosclerosis vs. Healthy Living Donor, *P*-value: 0.111. Membranous Glomerulonephropathy vs. Healthy Living Donor, *P*-value: 0.697. HLD, healthy living donor; MN, membranous glomerulonephropathy; FSGS, focal segmental glomerulosclerosis; MCD, minimal change disease.

For a long time, MCRs have been known to express in the kidney. The kidney is made up of a number of heterogeneous cell types, including vascular endothelial cells, glomerular mesangial cells, glomerular podocytes and parietal epithelial cells, tubular epithelial cells in different renal tubule segments, and renal interstitial cells. Unfortunately, to date, it has been barely clarified which MCR is expressed in what type of kidney cells. Recently, based on reverse transcription-polymerase chain reaction (RT-PCR) assay, Lindskog et al. (2010) demonstrated that MC1R is the major MCR expressed in human and rat kidneys, more specifically in podocytes, endothelial cells, mesangial cells, and tubular epithelial cells. RT-PCR may be a useful molecular biological technique, but it harbors potential pitfalls due to the subjective nature in data analysis and reporting as well as the technical limitations inherent in the assay like template quality and operator variability (Bustin and Nolan, 2004). As such, their initial results were likely unreliable, and not supported by their subsequent studies, in which cAMP induction was barely triggered in podocytes by selective MC1R agonists (Elvin et al., 2016). In a later study, this group posited that MC1R expression may be induced in podocytes upon stress or injury, despite very low or no expression of MC1R under physiological conditions. As such, they overexpressed human MC1R in cultured murine podocytes (Elvin et al., 2016) and confirmed that activation of MC1R by ACTH or by MC1R specific agonists was able to stimulate a cellular protective signaling cascade and protect the podocytes against injury. Nevertheless, somewhat contradictory to this finding, a number of other studies demonstrated that MC1R expression is predominant in renal tubules but very weak in glomeruli (Siegrist et al., 1994; Gatti et al., 2006; Lee et al., 2008; Botte et al., 2014). To determine the expression profile of MC1R in diseased kidneys, the web-based gene expression database and analysis platform for transcriptomic data of human kidney diseases (Nephroseq^1^, version Nephroseq v5) was employed. Shown in Figure 5, glomerular expression of MC1R is comparable and not statistically different between healthy living donors and renal patients with MN, FSGS or MCD, based on *post hoc* analysis of gene expression microarray data derived from the Nephroseq Ju CKD Glom dataset (Ju et al., 2013), suggesting that glomerular expression of MC1R, if any, is not augmented upon glomerular injury.

**FIGURE 6:**
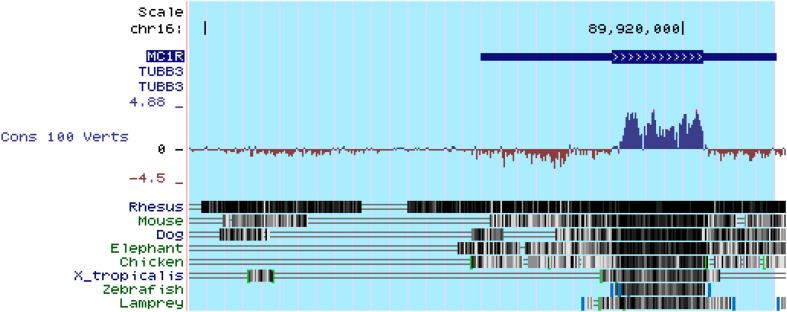
All of the five types of melanocortin receptors are encoded by intronless genes, as exemplified here by the *MC1R* gene. MC1R is located at chr16:89,914,847-89,920,951. As shown above in genomic annotation of MC1R with UCSC Genome Browser (GRCh38/hg38), MC1R is highlighted and there is no intron in MC1R. Comparative Genomics indicated that MC1R is highly conserved among different species (http://genome.ucsc.edu/).

In addition to MC1R, multiple other MCRs have also been reported to express in the kidney. Based on PCR amplification of human kidney-specific cDNA, Chhajlani and Wikberg (1996) found strong expression of MC5R and weak expression of MC2R in human kidney. Ni et al. (2006) found that MC3R, MC4R, and MC5R were expressed in rat kidney using the same method. It appears that these discrepant findings may reflect species differences, but may also be explained by potential pitfalls of the detection method, i.e., RT-PCR. Because all of the MCR genes are intronless (Figure 6), genomic contamination may confound the RT-PCR results. To address this issue, some studies prepared kidney mRNA in the presence of DNase and the quality was ascertained by the absence of amplicons of glyceraldehyde 3-phosphate dehydrogenase (GAPDH) introns. It turned out that rat kidneys mainly express MC1R and MC5R. However, MC1R is mainly located to renal tubules but very weakly expressed in glomeruli in rats (Si et al., 2013). Similar findings were also made in murine kidneys (Qiao et al., 2020), suggesting that MC1R is unlike a major mediator of the protective effect of melanocortins on glomeruli. In consistency, the glomerular protective and anti-proteinuric effect of NDP-MSH in murine models of podocytopathy elicited by LPS (Qiao et al., 2016) or Adriamycin (Qiao et al., 2020) was completely retained in mice with loss-of-function or null mutations in MC1R. Furthermore, in complementary clinical studies, patients with steroid-resistant nephrotic glomerulopathies, like idiopathic membranous nephropathy or focal segmental glomerulosclerosis, responded satisfactorily to ACTH monotherapy and ultimately achieved clinical remission, despite the dominant-negative mutation status of their MC1R gene as evidenced by the congenital red hair color and by gene sequencing (Qiao et al., 2016), suggesting that a steroidogenic-independent non-MC1R-mediated melanocortinergic signaling contributes to the beneficial effect of melanocortin therapy in glomerular disease. The exact identity of the MCR that mediates the glomerular protection warrants in-depth investigations. Since most glomerular diseases involve both glomerular cell autonomous injury and systemic immunopathogenic mechanisms, the melanocortinergic signaling driven by this MCR may have renal and systemic effects.

One of the critical physiological functions of the kidney is to maintain electrolyte homeostasis. This process is known to be heavily regulated by the melanocortin peptides, in particular γ-MSH. γ-MSH has a high affinity for MC3R and thereby regulates a variety of physiological activities, including energy homeostasis, food intake, hemodynamics, natriuresis and blood pressure regulation. MC3R is expressed in the brain and also abundantly in the heart and renal distal tubules (Humphreys, 2004). γ-MSH promotes urinary salt excretion via activating MC3R in distal renal tubules. In support of this, MC3R knockout or inhibition of γ-MSH production in mice caused salt-sensitive hypertension (Kathpalia et al., 2011).

## The Effect of Melanocortins on Kidney Disease

### Acute Kidney Disease (AKI)

Acute kidney injury (AKI) is a common and devastating complication in critically ill patients, characterized by an abrupt deterioration of kidney function, and associated with high morbidity and mortality. AKI is a complex syndrome with a variety of different etiologies and pathophysiological mechanisms. Most often, AKI is caused by ischemia or toxins and is referred to as acute tubular necrosis (ATN) pathologically (Kohda et al., 1998). There has been little improvement in the treatment of AKI despite hemodialysis and fluid replacement therapy in the past 30 years. So the demand for novel interventions of AKI is great. Animal models resembling human AKI has allowed scientists to effectively understand the pathogenic mechanisms and to aid in the research and development of prevention and treatment measures (Pippin et al., 2009). α-MSH, a melanocortin with the highest affinity for all MCRs (except for MC2R), has been reproducibly shown to effectively protect the kidney in pre-clinical models of AKI elicited by renal ischemia/reperfusion or by nephrotoxic drugs (Kolgazi et al., 2007; Doi et al., 2008).

Chiao et al., were the first to demonstrate that α-MSH is able to inhibit inflammation and protect against AKI (Lipton et al., 1994; Chiao et al., 1997). They found that α-MSH significantly reduced plasma blood urea nitrogen (BUN), creatinine levels, and improved mortality in rats and mice with renal ischemia/reperfusion injury elicited by renal artery clamping for 30 or 40 min. This was consistent with histologic evidence that α-MSH treatment significantly inhibited necrosis, sloughing, and obstruction of the proximal straight tubules, which were severely damaged in the ischemic kidney (Chiao et al., 1997). Further study also found effective renal protection from α-MSH in the isolated kidney where neutrophils were completely eliminated, thus suggesting a direct kidney protective effect of α-MSH (Chiao et al., 1998). In agreement, another study by Jo et al. (2001b) suggested that α-MSH could directly mitigate tubular cell apoptosis and thus may play a role in mediating the beneficial effect in ischemic AKI. In addition, Deng et al., found that α-MSH improved recovery of renal function in a model that mimics kidney transplantation (Deng et al., 2001). Moreover, Li et al. (2006) showed that α-MSH can prevent deterioration of renal function in obstructive nephropathy.

In order to test the possible effect of α-MSH in an animal model more relevant to human AKI, Simmons et al. (2010) employed the ischemia induced acute kidney injury in a porcine surgical model and found a robust renoprotective effect of AP214, an analog of α-MSH. Besides the ischemic AKI model, AP214 had been tested in mice with cecal ligation and puncture (CLP), a model of sepsis-induced AKI. Intravenous injection of AP214 improved blood pressure and heart rate in CLP mice, suggesting a beneficial effect of AP214 on hemodynamics. In addition, AP214 inhibited inflammation, improved kidney function, and reduced mortality in sepsis-induced AKI, even when AP214 therapy was started 6h after injury (Doi et al., 2008).

The efficacy of melanocortins was also tested in animal models of nephrotoxic AKI. Miyaji et al., examined the effect of α-MSH in murine models of mercuric chloride (HgCl_2_) induced AKI and found that α-MSH failed to reduce the level of serum creatinine and tubular damage. They posited that α-MSH may be more effective when the renal injury involves leukocyte-endothelial interactions rather than direct tubular toxicity (Miyaji et al., 2002). In contrast, in the study by Kolgazi et al., α-MSH exhibited a beneficial effect on gentamicin-induced nephrotoxicity and AKI, possibly via suppression of neutrophil infiltration and reactive oxygen metabolite induced lipid peroxidation (Kolgazi et al., 2007). Although these pre-clinical studies are promising, further research is still required to determine the usefulness of α-MSH in clinical settings in patients.

Although ACTH is also a potent agonist to all of the five MCRs, the effect of ACTH on AKI, unlike α-MSH, has been barely investigated. The only study by Si et al., examined the effect of ACTH in two models of AKI, namely tumor necrosis factor-induced AKI in rats and CLP-induced AKI in mice (Si et al., 2013). ACTH therapy demonstrated a remarkable protective effect in both models. Of note, the beneficial effect of ACTH on AKI continued to increase even when the level of corticosteroid reached its peak, suggesting both steroidogenic-dependent and -independent (α-MSH-like) mechanisms may contribute. As such, ACTH is likely superior to α-MSH in treating AKI, owing to its unique steroidogenic effect (Si et al., 2013).

### Nephrotic Glomerulopathy

A plethora of evidence supports the beneficial effect of melanocortins in glomerulopathy (Table 2), including membranous nephropathy (MN), minimal change disease (MCD), focal segmental glomerular sclerosis (FSGS), and immunoglobulin A nephropathy (IgAN) (Gong, 2014).

**TABLE 2:** The effect of melanocortins in kidney disease.

Kidney Diseases		References	Models/Diseases	Effect of melanocortin treatment
AKI		Chiao et al., 1997	Mice and rats with renal ischemia/reperfusion	Inhibits inflammation (neutrophils, chemokines), NO; attenuates necrosis, sloughing and obstruction of the proximal straight tubules; reduces BUN, Scr, and mortality
		Chiao et al., 1998	ICAM-1 knock-out mice with renal ischemia/reperfusion; isolated mouse kidneys	Reduces BUN, renal cortex necrosis, and NO
		Jo et al., 2001b	Rats with renal ischemia/reperfusion	Improves tubular-cell apoptosis; reduces BUN, Scr, tubular necrosis and tubular obstruction; inhibits inflammation
		Deng et al., 2001	Rat kidney transplantation model	Increases recovery of renal function
		Li et al., 2006	Rats with bilateral ureteral obstruction	Attenuates the downregulation of AQP2, AQP3, Na-K-ATPase; reduces renal tubular cell apoptosis; improves GFR
		Simmons et al., 2010	Pigs with left nephrectomy and ischemia of the right kidney	Reduces Scr; increases eGFR
		Doi et al., 2008	Mice with septic AKI induced by cecal ligation and puncture	Improves blood pressure and heart rate; inhibits inflammation; improves kidney function; and reduces mortality
		Miyaji et al., 2002	Mice with mercuric chloride-induced AKI	Fails to reduce the level of serum creatinine and tubular damage
		Kolgazi et al., 2007	Rats with gentamicin-induced AKI	Reduces the severity of renal histological damage; fails to restore the impaired renal function.
		Si et al., 2013	Rats with TNF-elicited AKI; Mice with septic AKI induced by cecal ligation and puncture	Improves survival and eGFR; reduces proteinuria, tubulointerstitial injury score, vacuolization area, dilation/sloughing and tubular cell apoptosis
Nephrotic glomerulopathy	MN	Elvin et al., 2016	Rats with passive Heymann nephritis	Restores serum albumin levels; improves proteinuria, glomerular morphology, podocyte injury; reduced oxidative stress, urine TBARS
		Hladunewich et al., 2014	Patients with iMN	Maintains renal function; reduces proteinuria; 60% (12/20) of patients achieve complete or partial remission of proteinuria
		van de Logt et al., 2015	Patients with iMN	Reduces proteinuria; 55% (11/20) of patients attain complete or partial remission of proteinuria
		Kittanamongkolchai et al., 2016	iMN patients resistant to other immunosuppressants	Reduces proteinuria; 86% (25/29) of patients attain complete or partial remission of proteinuria
	MCD or FSGS	Lindskog Jonsson et al., 2014	Mice with Adriamycin-induced podocytopathy	No statistical significance in albuminuria, degree of foot process effacement, disrupted glomerular structures after melanocortin therapy
		Qiao et al., 2016, 2020	Mice with Adriamycin or LPS-induced podocytopathy	NDP-MSH prominently improved proteinuria, glomerular damage, podocyte ultrastructure via an MC1R-independent mechanism
		Filippone et al., 2016	FSGS or MCD patients resistant to conventional therapy	All three patients with MCD achieved complete and partial remission; 40% (4/10) of FSGS patients attained complete and partial remission
		Hogan et al., 2013	Patients with refractory FSGS	29% (7/24) of patient achieved complete or partial remission, of which 5 had steroid resistance
		Madan et al., 2016	Patients with refractory FSGS	2/2 patients achieved complete or partial remission
		Wang et al., 2017	Pediatric patients with frequently relapsing or steroid-dependent nephrotic syndrome	Ineffective at preventing disease relapses in pediatric nephrotic syndrome (14/15 relapsed on ACTH treatment)
	IgAN	Prasad et al., 2018	Patients with refractory IgAN	1 patient achieved complete remission after ACTH monotherapy; and ACTH was prescribed as a steroid-sparing agent in combination with cyclophosphamide for the other 2 patients
		Bomback et al., 2012	Patients with refractory IgAN	One patient achieved complete remission in ACTH monotherapy
		Bomback et al., 2011	Patients with refractory IgAN	Two patients with steroid-resistant IgAN demonstrated 50% reductions in proteinuria
	DN	Tumlin et al., 2013	Patients with DN	57% (8/14) patients achieved complete or partial remission as a long-term effect.
		Madan et al., 2016	Patients with DN	1 patient showed ≥ 30% proteinuria reduction; 2 had no response and 1 end up in early termination
	LN	Botte et al., 2014	Murine lupus-like models	Reduces glomerular IgG deposits and reduces lupus activity
		Bomback et al., 2012	Patients with LN	Patients with LN (class V) showed no response to ACTH treatment
	Other glomerular diseases	Berg and Arnadottir, 2004	Patients	6 MsPGN, 1 MCGN, 1 hereditary nephritis achieved complete or partial remission
		Bomback et al., 2011	Patients	1 monoclonal DPGN patient failed to respond to ACTH therapy
Interstitial nephritis and kidney fibrosis		Jo et al., 2001a	cultured human renal tubular cells	α-MSH treatment significantly reduced CsA-induced cellular apoptosis
		Lee et al., 2004	Rats with CsA nephrotoxicity	Improves renal cell apoptosis, inflammation and tubulointerstitial fibrosis

*AKI, acute kidney disease; NO, nitric oxide; ICAM, intercellular adhesion molecule; AQP, aquaporin; CLP, cecal ligation and puncture; TBARS, thiobarbituric acid-reactive substances; MsPGN, mesangioproliferative glomerulonephritis; MCGN, mesangiocapillary glomerulonephritis; DPGN, diffuse proliferative glomerulonephritis; CsA, cyclosporine A; TNF, tumor necrosis factor; MN, membranous nephropathy; MCD, minimal change disease; FSGS, focal segmental glomerular sclerosis; LPS, lipopolysaccharides; NDP-MSH, [Nle4, DPhe7]-α-melanocyte-stimulating hormone; IgAN, immunoglobulin A nephropathy; DN, diabetic nephropathy; LN, lupus nephritis.*

#### MN

A number of melanocortins, including ACTH, α-MSH and the MC1R agonist MS05, have been tested in rats with Passive Heymann nephritis (PHN), a classical model of human MN (Pippin et al., 2009). Both α-MSH and MS05 significantly reduced proteinuria and improved glomerular injury. ACTH therapy had a tendency to reduce the level of proteinuria, but it did not reach statistical significance, possibly due to insufficient dosing or lack of statistical power (Lindskog et al., 2010). Subsequently, in the clinical trial by Hladunewich et al., patients with biopsy proven idiopathic MN received ACTH gel monotherapy, and 60% (12 out of the 20 patients) achieved a complete or partial remission (Hladunewich et al., 2014). The synthetic ACTH was also tested in another study and showed a similar efficacy with a 55% complete and partial remission rate (11 out of 20 patients) (van de Logt et al., 2015). A meta-analysis of the efficacy of ACTH in the treatment of glomerular diseases was carried out and revealed an 86% (25 out of 29 patients) remission rate for patients with iMN, who were converted to ACTH monotherapy after having failed other immunosuppressive therapy including glucocorticoids (Kittanamongkolchai et al., 2016). In view of the newly discovered pathogenic mechanism underlying iMN in men that involves the autoantibodies against anti-phospholipase A2 receptor (PLA2R), several studies examined the longitudinal response of the anti-PLA2R antibodies to ACTH treatment in iMN patients, and found that the titers of anti-PLA2R antibodies decreased prior to proteinuria remission, entailing a potential role of ACTH in inducing immunological remission in iMN. Because most patients had been resistant to corticosteroids or the Ponticelli regimen, this immunosuppressive effect of ACTH cannot be explained by the steroidogenic dependent pathway, but is more likely mediated directly through the MCRs expressed in immune cells (Gong, 2014).

#### MCD and FSGS

In experimental models of podocytopathy (LPS or Adriamycin-induced nephropathy), Qiao et al., found that NDP-MSH therapy protected against podocyte injury and glomerular damage and ameliorated proteinuria (Lindskog Jonsson et al., 2014). In agreement in clinical settings, ACTH demonstrated great efficacy as a valuable alternative choice to treat FSGS and/or MCD for patients intolerant or resistant to conventional therapies, including corticosteroids (Filippone et al., 2016). In support of this, ACTH therapy successfully induced complete or partial remission in 7 out of 24 FSGS patients (29%) who had previously failed other immunosuppressive regimens (Hogan et al., 2013). Of these 7 patients, 5 were steroid resistant and 2 were steroid dependent. There is little data on the effect of melanocortin therapy in MCD, but two case series, respectively, involving 2 and 3 patients with MCD showed great remission rate after ACTH therapy (Filippone et al., 2016; Madan et al., 2016).

#### IgA Nephropathy

While IgA nephropathy is a common cause of chronic kidney disease in some Asian countries, the published data regarding the effectiveness of melanocortins in this population is little except for several case series reports (Wyatt and Julian, 2013). Recently, Prasad et al. (2018) found that the use of synthetic ACTH, either as monotherapy or as a steroid-sparing agent, achieved excellent outcomes in IgAN, and in most cases induced complete proteinuria remission. This is consistent with the experience of using natural ACTH in patients with IgAN in the United States (Bomback et al., 2011, 2012; Prasad et al., 2018).

#### Diabetic Nephropathy (DN)

Diabetic nephropathy is the leading cause of chronic kidney disease (CKD) in Western societies, and one of the serious complications of diabetes mellitus with no definite therapy available yet (Webster et al., 2017). So far, the effect of melanocortins on DN has been barely examined by pre-clinical studies. In terms of clinical trials, due to the long-standing controversy over the therapeutic benefit versus the risk of adverse effect of corticosteroids in diabetes associated kidney diseases, very few have been done to evaluate the effect of ACTH in DN. Tumlin et al., performed the first randomized open-label pilot trial to test the effect of low-dose ACTH in DN (Jauregui et al., 2009; Leeuwis et al., 2010; Tumlin et al., 2013). Their results showed that ACTH treatment achieved complete or partial remission in 57% (8 out of 14 patients) of the patients. No significant difference in the efficacy of reducing proteinuria was detected between the daily 16 IU dose group and the 32 IU dose group (Tumlin et al., 2013). Another study done by Madan et al. (2016) involved only 4 DN patients, of which one patient showed ≥ 30% proteinuria reduction, and the rest either had no response or ended the study early due to side effects. Collectively, clinical evidence suggests that melanocortin therapy might be useful in the treatment of DN, but large scale clinical trials are warranted to test the exact efficacy.

#### Lupus Nephritis (LN)

Systemic lupus erythematosus (SLE) is an autoimmune disorder that affects many organ systems with one of the most common and serious complications being LN. Several case reports suggested a possible benefit of ACTH therapy in patients with LN (Bomback et al., 2011; Li et al., 2015). On the contrary, in a murine lupus-like model induced by pristane, NDP-MSH treatment did not reduce proteinuria or albuminuria, but it did improve histological markers of renal injury like glomerular IgG deposition and did reduce lupus activity, marked by reduction in hypergammaglobulinemia, anti-nuclear antibodies, and anti-neutrophil cytoplasmic plasma antibodies (Botte et al., 2014). The mechanism responsible for this discrepancy was not fully understood. But a retrospective study to evaluate the role of ACTH in SLE treatment suggested ACTH as an invaluable alternative to corticosteroids in the treatment of SLE. ACTH appears to be safe and well-tolerated after 6 months of treatment, with a significant reduction in lupus activity (Li et al., 2015).

#### Other Glomerular Disease

Based on some case series reports, ACTH treatment seems to be effective in a number of other glomerular diseases, including mesangioproliferative glomerulonephritis (MsPGN), mesangiocapillary glomerulonephritis (MCGN), and hereditary nephritis, though pre-clinical evidence is still lacking (Berg and Arnadottir, 2004; Bomback et al., 2011).

### Progressive CKD

Regardless of the original etiology, the final common pathway for the progression of CKD is kidney fibrosis, characterized by glomerulosclerosis, tubular atrophy, inflammation, and interstitial fibrosis (Webster et al., 2017). The effect of melanocortins on progressive CKD has been explored in pre-clinical studies by using *in vivo* or *in vitro* models of renal interstitial injury and fibrosis, including the model of Cyclosporine A (CsA) nephropathy, which recapitulates key features of renal tubular atrophy and interstitial fibrosis elicited by the use of CsA in human patients with high fidelity (Shihab et al., 1999). Jo et al. (2001a) found that α-MSH treatment significantly attenuated CsA-induced apoptosis in cultured human tubular cells. *In vivo* in the rat model of CsA nephrotoxicity, Lee et al. (2004) demonstrated that α-MSH can mitigate the CsA-induced tubulointerstitial fibrosis as well as tubular cell apoptosis. The results of these two studies may pave the way to expand the clinical indications of melanocortin therapy.

## Side Effects

All data to date has shown that the adverse effects of melanocortins, in particular ACTH, are mild, tolerable, and reversible, though it was commonly mentioned that ACTH therapy may cause corticosteroid-like side effects. In contrast, patients treated with glucocorticoids experienced more debilitating side effects including infection, hypertension, glucose intolerance, obesity, sleep disorders and others (Gong, 2014). Of note, most melanocortins are peptides or peptide derivatives and thus are biological macromolecules, which is inevitably antigenic and may trigger immune reactions. Indeed, the use of animal-derived natural ACTH to treat glomerular disease has been associated with *de novo* formation of neutralizing antibodies in some sensitive patients, followed by an acquired resistance to ACTH therapy (Wang et al., 2017; Shrivastava et al., 2020). As such, there is a pressing need to develop small molecule MCR agonists or melanocortin analogs with less immunogenicity for improving the therapeutic efficacy in patients with kidney diseases.

## Conclusion

The melanocortin hormone system is a complex and incompletely understood neuroimmunoendocrine circuitry in the mammalian body. The physiological interaction of its constituent components increases the complexity of this hormone system. In recent years, new understandings about the mechanisms of action of melanocortins promoted tremendous exploration in this field. There has been a lot of evidence showing that melanocortins confer renoprotective effects in animal models and in humans. Much of the compelling clinical evidence is obtained from the use of ACTH in patients with steroid-resistant glomerular disease. As a typical melanocortin peptide, ACTH protects the kidney through at least two mechanisms: (1) stimulating the production of corticosteroids; and (2) activating MCRs expressed by diverse kidney parenchymal cells. The latter one still needs continued research, considering the complexity of the types of kidney cells, and the cross-interaction between MCRs. However, with the advances in developing more specific synthetic melanocortins and with the application of transgenic animals with genetic ablation of specific components of the melanocortin system, it is believed that much progress will be made in the near future regarding the role of melanocortinergic pathways in kidney pathobiology.

## Author Contributions

RG devised the conceptual ideas. MC performed the research. LD contributed to discussion. MC and RG wrote the manuscript. BC and JS contributed to revision of the manuscript. All authors approved the final version of the manuscript.

## Conflict of Interest

RG and LD report research funding from the Mallinckrodt Pharmaceuticals. RG served as a consultant to the Questcor Pharmaceuticals and the Mallinckrodt Pharmaceuticals. The remaining authors declare that the research was conducted in the absence of any commercial or financial relationships that could be construed as a potential conflict of interest.

## Footnotes

1 www.nephroseq.org
